# Unveiling the Catalytic Potential of Topological Nodal-Line Semimetal AuSn_4_ for Hydrogen Evolution and CO_2_ Reduction

**DOI:** 10.1021/acs.jpclett.3c00113

**Published:** 2023-03-22

**Authors:** Danil W. Boukhvalov, Gianluca D’Olimpio, Federico Mazzola, Chia-Nung Kuo, Sougata Mardanya, Jun Fujii, Grazia Giuseppina Politano, Chin Shan Lue, Amit Agarwal, Ivana Vobornik, Piero Torelli, Antonio Politano

**Affiliations:** † College of Science, Institute of Materials Physics and Chemistry, Nanjing Forestry University, Nanjing 210037, P. R. China; ‡ Institute of Physics and Technology, Ural Federal University, Mira Str. 19, 620002 Yekaterinburg, Russia; § Department of Physical and Chemical Sciences, University of L’Aquila, via Vetoio, 67100 L’Aquila (AQ), Italy; ∥ Consiglio Nazionale delle Ricerche (CNR), Istituto Officina dei Materiali (IOM), Laboratorio TASC, Area Science Park S.S. 14 km 163.5, 34149 Trieste, Italy; ⊥ Department of Physics, National Cheng Kung University, 1 Ta-Hsueh Road, 70101 Tainan, Taiwan; ¶ Department of Information Engineering, Infrastructures and Sustainable Energy (DIIES), University “Mediterranea” of Reggio Calabria, Loc. Feo di Vito, 89122 Reggio Calabria, Italy; ∇ Department of Physics, Indian Institute of Technology Kanpur, Kanpur 208016, India

## Abstract

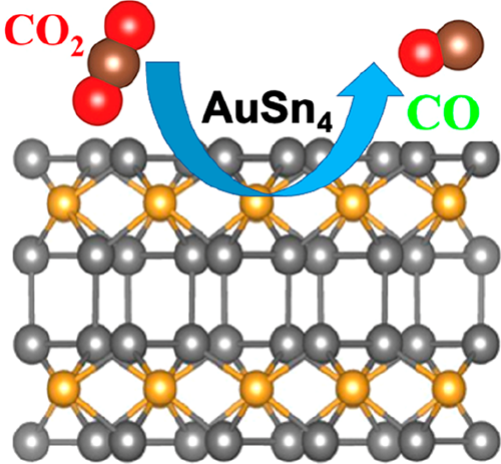

In recent years, the correlation between the existence of topological electronic states in materials and their catalytic activity has gained increasing attention, due to the exceptional electron conductivity and charge carrier mobility exhibited by quantum materials. However, the physicochemical mechanisms ruling catalysis with quantum materials are not fully understood. Here, we investigate the chemical reactivity, ambient stability, and catalytic activity of the topological nodal-line semimetal AuSn_4_. Our findings reveal that the surface of AuSn_4_ is prone to oxidation, resulting in the formation of a nanometric SnO_2_ skin. This surface oxidation significantly enhances the material’s performance as a catalyst for the hydrogen evolution reaction in acidic environments. We demonstrate that the peculiar atomic structure of oxidized AuSn_4_ enables the migration of hydrogen atoms through the Sn–O layer with a minimal energy barrier of only 0.19 eV. Furthermore, the Volmer step becomes exothermic in the presence of Sn vacancies or tin-oxide skin, as opposed to being hindered in the pristine sample, with energy values of −0.62 and −1.66 eV, respectively, compared to the +0.46 eV energy barrier in the pristine sample. Our model also suggests that oxidized AuSn_4_ can serve as a catalyst for the hydrogen evolution reaction in alkali media. Additionally, we evaluate the material’s suitability for the carbon dioxide reduction reaction, finding that the presence of topologically protected electronic states enhances the migration of hydrogen atoms adsorbed on the catalyst to carbon dioxide.

Quantum materials have attracted considerable attention in the past decade for the exotic phenomena enabled by their topologically nontrivial states.^1−5^ While most existing investigations are still focused on fundamental physics, it is well recognized that the goal of this vast scientific community is to (i) identify and (ii) implement technological applications, possibly scalable at the industrial level, exploiting the quantum phenomena associated with the topological protection of their electronic states. In recent years, topological materials have been proposed for applications in various fields, such as energy,^6^ optoelectronics,^7^ electronics,^8^ and recently topological catalysis.^9−11^ Especially, catalysis with quantum materials represents a new promising route in electrochemistry, considering that topological surface states mediate the charge transfer between the substrate and the adsorbed chemical species. Furthermore, the high carrier mobility associated with massless Fermions in topologically protected surface states with linear dispersion is beneficial for fast electron transfer. Accordingly, topological materials, naturally exhibiting (i) high electrical conductivity, (ii) nontrivial topologically protected surface states, and (iii) ultrahigh mobility charge carriers,^9,12,13^ represent promising candidates for the development next-generation catalysts. Similarly, PtGa,^14^ PtAl,^14^ TaP,^15^ NbP,^15^ TaAs,^15^ and NbAs^15^ have been proposed as catalysts for the hydrogen evolution reaction (HER).

Recently, PtSn_4_,^16^ PdSn_4_,^17,18^ and AuSn_4_^19,20^ have been reported to display an exotic structure of Dirac node arcs, which can be categorized in a new class of topological materials, namely, topological nodal-line semimetals. Especially, AuSn_4_ deserves particular attention, owing to the discovery of superconductivity,^12,21^ strictly correlated with the presence of Dirac-cone electrons in topological surface states.

In contrast to van der Waals layered materials, in AuSn_4_ the break of the metallic interlayer bonds originates in the topological surface states. Its chemical instability can be attributed to the formation of these broken bonds.^22−24^ Therefore, to assess the catalytic properties of topological materials, it is necessary to consider the surface oxidation and its relationship with the topologically nontrivial states. In this study, we aim to elucidate the correlation between catalysis and surface chemical reactivity in topological nodal-line semimetal AuSn_4_.

Using surface-science spectroscopies and density functional theory (DFT), we demonstrate that the catalytic activity is driven by the self-assembled SnO_2_/AuSn_4_ heterostructure formed upon interaction of the AuSn_4_ surface with ambient atmosphere. The diffusion of hydrogen atoms through the tin-oxide skin has a barrier of only 0.19 eV. Moreover, the Volmer step is energetically favorable in the presence surface oxidation (−1.66 eV), in contrast to the pristine surface. Moreover, both pristine and oxidized AuSn_4_ surfaces represent suitable catalysts for the carbon dioxide reduction reaction, as the presence of topologically protected electronic states favor the migration of the hydrogen atom adsorbed on the catalyst toward carbon dioxide, with the following conversion of the noncovalently adsorbed CO_2_ to −COOH or carboxyl groups.

AuSn_4_ belongs to the space group *Aba*2 (No. 41), and its atomic structure has alternating Au and Sn layers with a Sn-terminated surface (Figure 1a,b). Despite the fact that O contamination is often observed in the bulk crystals of Sn-based alloys,^25^ survey spectra obtained using X-ray photoelectron spectroscopy (XPS) (Figure 1c) reveal the absence of any such contamination in the bulk crystal. The crystal is oriented along a preferential (002) cleavage plane, as evidenced by the room-temperature X-ray diffraction (XRD) pattern (Figure 1d). The lattice constants evaluated from XRD are *a* = 6.497 ± 0.002 Å, *b* = 6.527 ± 0.002 Å, and *c* = 11.717 ± 0.002 Å, congruently with previous reports.^12^ The single-crystal XRD pattern (Figure 1d) demonstrates that the basal plane of a cleaved crystal is perpendicular to the *b*-axis. Figure 1e shows the theoretical band structure along the high symmetry direction X-Γ-X (see the inset to Figure 1e for the Brillouin zone), with spectral weight projected for several k_*z*_ values. The theoretical band structure was experimentally validated by synchrotron-based angle-resolved photoelectron spectroscopy (ARPES, Figure 1f), which confirmed the occurrence of complex multibands system. Especially, the band structure (Figure 1e–f) shows a metallic character of the samples, with prominent intensity given by electron-like pockets located at the center of the Brillouin zone. These “pockets” correspond to the appearance of the peaks on the Fermi level in the density of states (DOS, Figure 2). One of the peculiarities of topological materials, also observed in AuSn_4_, is the absence of a clear separation between the orbitals of tin and gold in the valence band. Note that in contrast to topological materials with covalent bonds, in AuSn_4_ gold and tin layers are held by metallic bonds, and therefore Au 5d and Sn 4d bands are overlapped (Figure 1).

**Figure 1:**
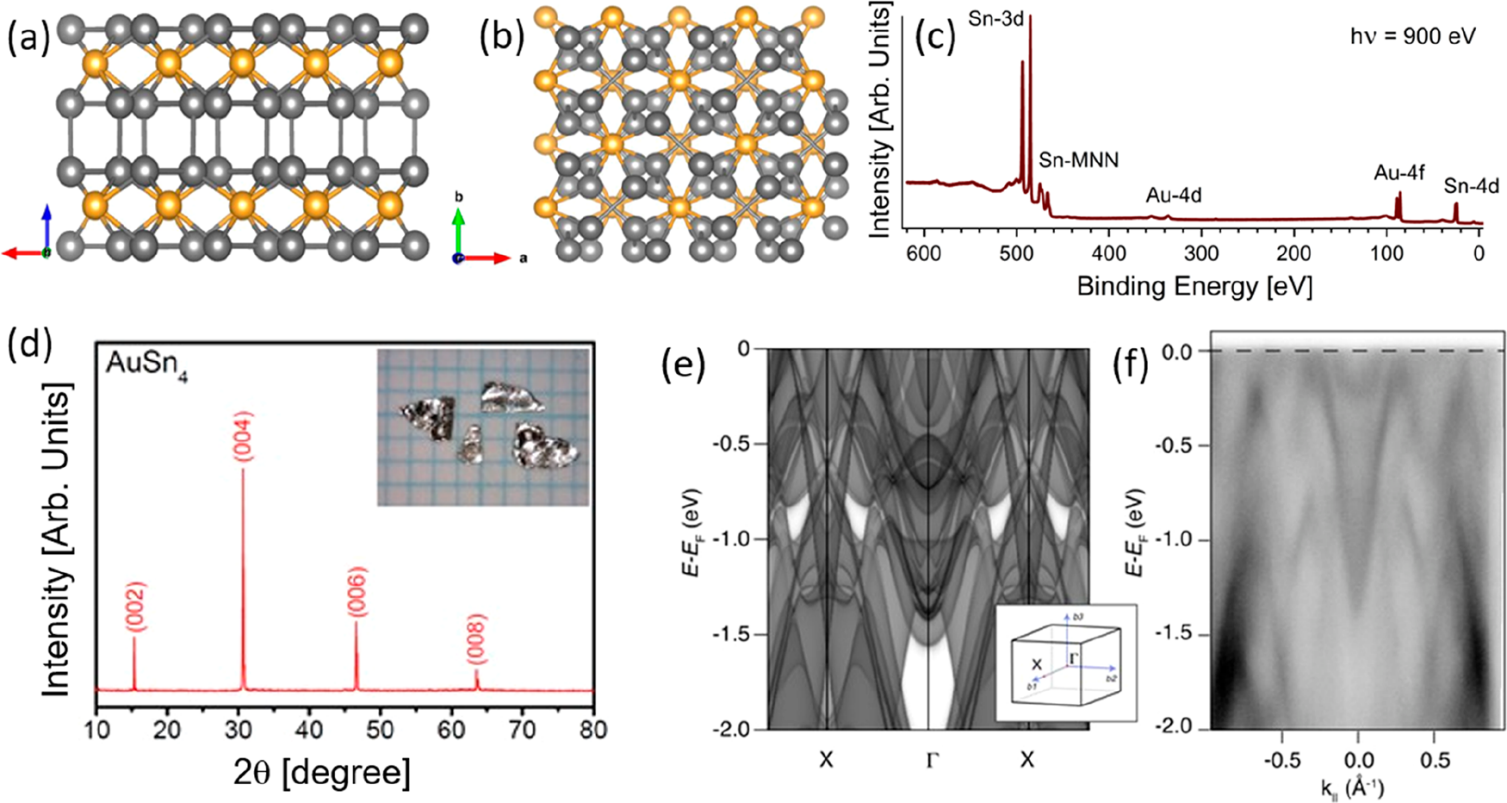
(a, b) Side and top views of the atomic structure of AuSn_4_. Yellow and gray balls denote Au and Sn atoms, respectively. (c) XPS survey spectrum of as-cleaved AuSn_4_. (d) XRD spectrum for a single crystal of AuSn_4_. The inset shows a photograph of grown single crystals. (e) Bulk electronic structure of AuSn_4_ obtained by density functional theory. Bands were obtained by projecting the available k_*z*_ within the whole Brillouin zone. (f) The corresponding ARPES data are shown as a sum of in-plane and out-of-plane light polarization, with photons of 48 eV. At this energy, it is possible to access the full Brillouin zone along Γ-X, along the orange line of the inset, and the photoemission matrix elements are favorable. The overall agreement between the calculated and the measured energy-momentum dispersion is evident.

**Figure 2:**
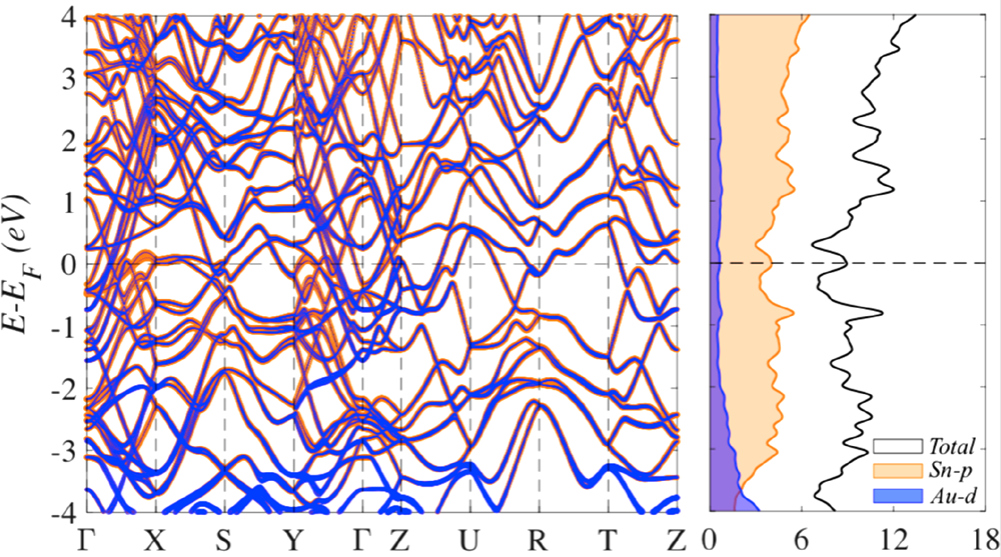
Orbital-resolved band structure (left) and total and partial densities of states of bulk AuSn_4_ (right).

To evaluate the chemical stability of the AuSn_4_ surface, we modeled physisorption and further decomposition of ambient gases on AuSn_4_. To account for defects naturally occurring in real samples, we modeled AuSn_3.88_, by including one Sn vacancy in the surface layer of the supercell (Figure 3a). Note that the calculated formation energy of a single Sn vacancy on the surface layer is 0.84 eV/Sn (81.2 kJ/mol). Thus, the amount of the vacancies in the surface layer is non-negligible.

**Figure 3:**
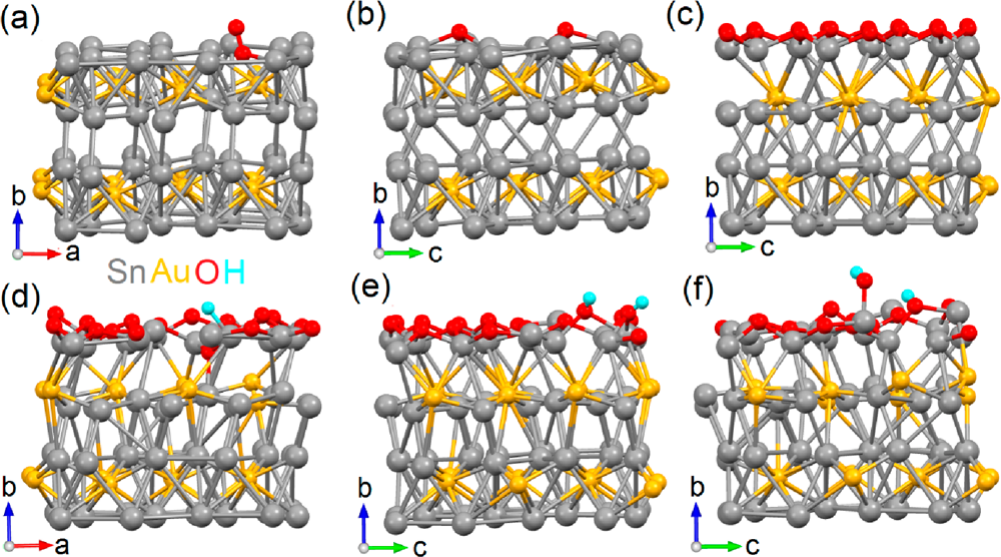
Optimized atomic structure of (a) molecular oxygen physisorbed on one Sn vacancy in the surface layer of AuSn_3.88_, (b) oxygen molecule decomposed on the surface of AuSn_4_, (c) formation of oxidized layer on the surface of AuSn_4_. Panels d–f represent the various reactions steps of the HER on the oxidized surface of AuSn_4_, namely (d) adsorption of hydrogen, (e) decomposition of water, and (f) first step of desorption of OH^–^ on the oxidized surface of AuSn_4_.

In Table 1 the differential enthalpy and Gibbs free energy of physisorption at room temperature of carbon monoxide, water, and molecular oxygen are reported. Physisorption of carbon monoxide and water on a defect-free surface of AuSn_4_ at room temperature is energetically unfavorable, as indicated by the positive value of the differential Gibbs free energy of physisorption (Δ*G* = +11.33 kJ/mol, Δ*G* = +30.3 kJ/mol, respectively). At room temperature, AuSn_4_ is reactive only toward molecular oxygen (Δ*G* = −36.8 kJ/mol).

**Table 1:** Differential Enthalpy for Physisorption Δ*H*_ads_, Differential Gibbs Free Energy at Room Temperature for Physisorption Δ*G*, and Differential Enthalpy of Decomposition Δ*H*_dec_ (all in kJ/mol) of Selected Molecules over the Defect-free Surface of AuSn_4_ and the Defective Surface of AuSn_3.88_

		physisorption		
substrate	molecule	Δ*H*_ads_ [kJ/mol]	Δ*G* [kJ/mol]	decomposition Δ*H*_dec_ [kJ/mol]
AuSn_4_	CO	–8.22	+11.33	+430.29
	H_2_O	–0.09	+30.31	–36.33
	O_2_	–48.29	–36.8	–431.06 (−373.13)
AuSn_3.88_	CO	–54.13	–34.78	+130.51
	H_2_O	–136.42	–105.12	+158.79
	O_2_	–210.23	–198.74	–276.04 (−369.19)

On the other hand, the introduction of defects on the surface leads to a negative value of Δ*G* for all the considered ambient gases. In particular, Figure 3a shows the optimized atomic structure of molecular oxygen physisorbed on the Sn vacancy in the surface layer of AuSn_3.88_. It should be noted that, although CO adsorption is energetically favorable in AuSn_3.88_ (Δ*G* = −34.79 kJ/mol), physisorption of molecular oxygen and water is largely preferential (Δ*G* values of −105.12 and −198.74 kJ/mol, respectively), so that the Sn-vacancy sites will be occupied before by O_2_ and H_2_O. Hence, AuSn_4_-based electrodes can be considered as CO-tolerant.

Physisorption might be the first step toward decomposition of adsorbed molecules. To evaluate the possibility of this scenario, the differential enthalpy of decomposition Δ*H*_dec_ for water and molecular oxygen was calculated (values in Table 1, see Figure 3b for a visualization).

Water decomposition is energetically favorable only on the defect-free AuSn_4_ surface (Table 1). One should consider that Δ*H*_dec_ for water was calculated considering the adsorption of water from air. However, in the case of water adsorption from liquid, the contribution from entropy should be smaller, since the energy of noncovalent interactions of water with AuSn_4_ is smaller than the energy of hydrogen bonds with other water molecules. Therefore, spontaneous decomposition of water in liquid state could occur.

Since the decomposition energy of oxygen molecules is quite high (above 200 kJ/mol), we opted to simulate the oxidation of the entire surface (Figure 3c). The computational results (Table 1) clearly demonstrate that this process is highly energetically favorable for both of the substrates examined, with a value of approximately −370 kJ/mol. Therefore, similarly to other transition-metal stannides, such as PtSn_4_^22^ and PdSn_4_,^10^ the surface of AuSn_4_ will be immediately oxidized. The chemical instability of the surface layer is caused by a combination of factors, including the presence of broken Sn–Sn interlayer bonds that occur during the formation of the Sn-terminated surface, as well as charge transfer between surface Sn and subsurface Au atoms (as shown in Figure 4a). Although the formation of the surface does not result in any additional peaks near the Fermi level (as indicated in Figure 4c), we propose that the second factor—the charge transfer—plays a more significant role in the chemical instability of the surface.

**Figure 4:**
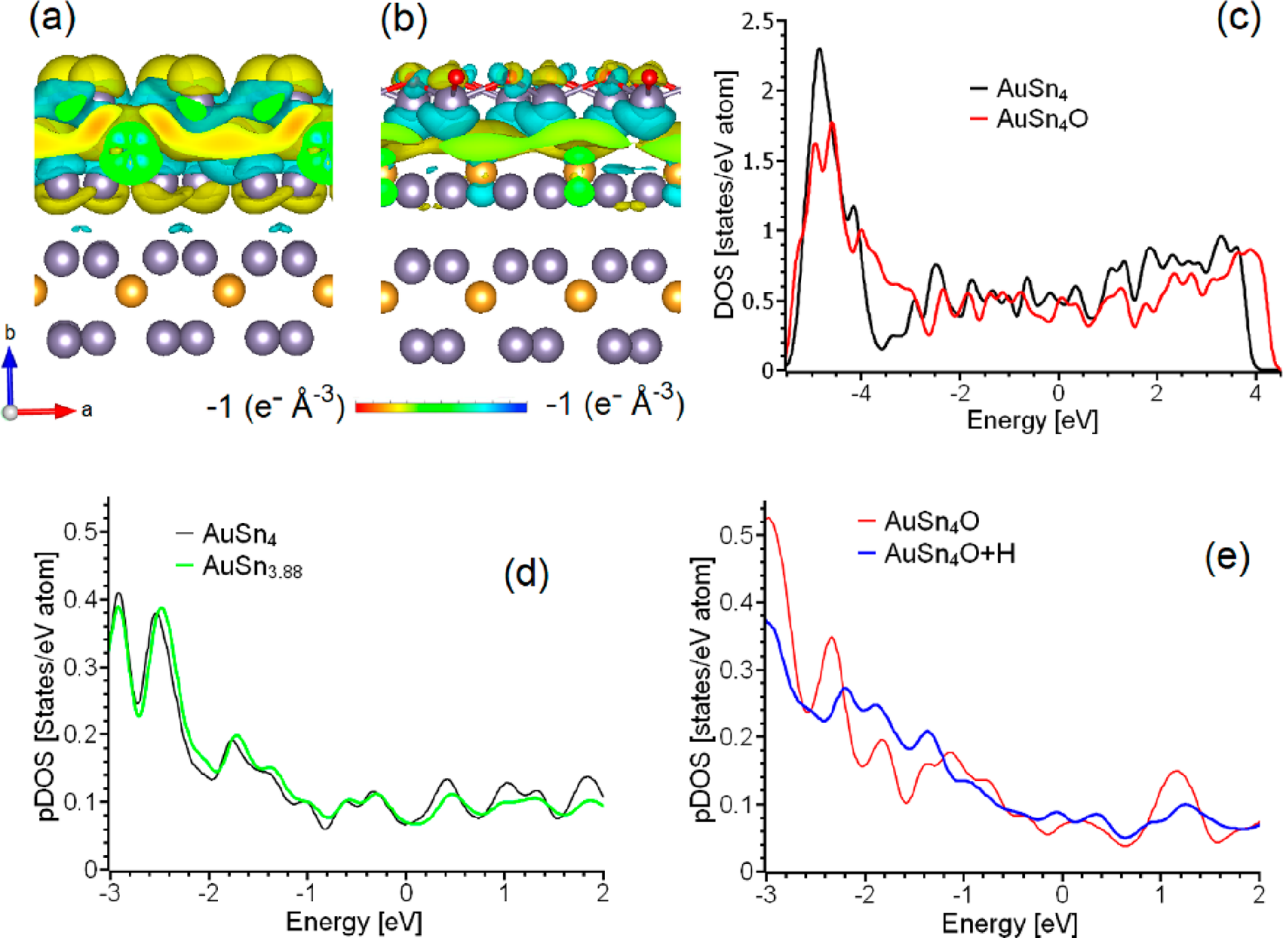
Changes of the charge density after formation of the interface between (a) surface Sn and (b) SnO_2_ layers and the subsurface part of the AuSn_4_ slab. Panel (c) reports the total densities of states for both structures shown on panels (a) and (b). Panels (d) and (e) report the partial densities of states (pDOS) of 5d orbitals of the Au atoms from subsurface layers for the cases of (d) AuSn_4_ and AuSn_3.88_ (in the latter case, the pDOS is shown for the Au atom closest to Sn vacancy), and for (e) AuSn_4_O and AuSn_4_O+H (in the latter case, the pDOS is shown for the Au atom closest to adsorbed H).

The formation of an oxidized layer induces both quantitative changes in the doping of the surface layer from the subsurface region and visible modifications in the electronic structure of the subsurface Au atomic layer (Figure 4d vs Figure 4e). The contribution of Au 5d-states to the topological features in the band structure near the Fermi level is significant (blue lines in Figure 2). Therefore, the oxidation of the Sn surface layer destroys the topological features in the Sn–Au–Sn trilayer. The changes in the electronic structure of the subsurface layer arise not only from charge redistribution, but also from the displacement of the Au atoms from their stoichiometric positions. In the bulk crystal layer, the Au atoms are precisely located between two Sn layers (as shown in Figure 1). However, the formation of the surface with the breakage of Sn–Sn interlayer bonds leads to a shift of the Au layer away from the surface (Figure 3a,b). This alteration in the symmetry of the subsurface layers results in a deviation of the electronic structure of the AuSn_4_ slab from that of the bulk (Figure 3c,d vs Figure 2). Additionally, the oxidation of the entire Sn surface layer further displaces the Au layer into the bulk (Figure 3c). Therefore, changes in the electronic structure of subsurface layers are not only caused by the passivation of surface dangling bonds, but also by variations in the atomic structure of the subsurface layers.

The theoretical model was validated by experiments on the surface chemical reactivity of AuSn_4_ by X-ray photoelectron spectroscopy (XPS), using a synchrotron light source to optimize the surface sensitivity and energy resolution. High-resolution XPS spectra of Au-4f, Sn-3d, and O-1s core levels for the as-cleaved AuSn_4_ and the same surface modified by the exposure to 10^4^ L (1L = 10^–6^ Torr s^–1^) of O_2_, H_2_O, and CO are reported in Figure 5. Specifically, the Au-4f core level was recorded at a binding energy (BE) of 85.0 (*J* = 7/2) and 88.6 (*J* = 5/2) eV, congruently with other Au–Sn alloys.^26,27^ The spin–orbit split doublet was practically unchanged upon O_2_, H_2_O, and CO exposure. Correspondingly, the Sn-3d core level for as-cleaved AuSn_4_ was recorded at a BE of 485.0 (*J* = 5/2) and 493.4 (*J* = 3/2) eV.^26^ Upon O_2_ exposure, two additional components arising from SnO and SnO_2_ appeared in Sn-3d core levels, with the *J* = 5/2 components at 486.0 and 486.6 eV,^22,28^ respectively, with intensities as high as 26% and 27% of the total spectral area. Similarly, H_2_O dosage on AuSn_4_ provided the same oxide components, with similar intensity.

**Figure 5:**
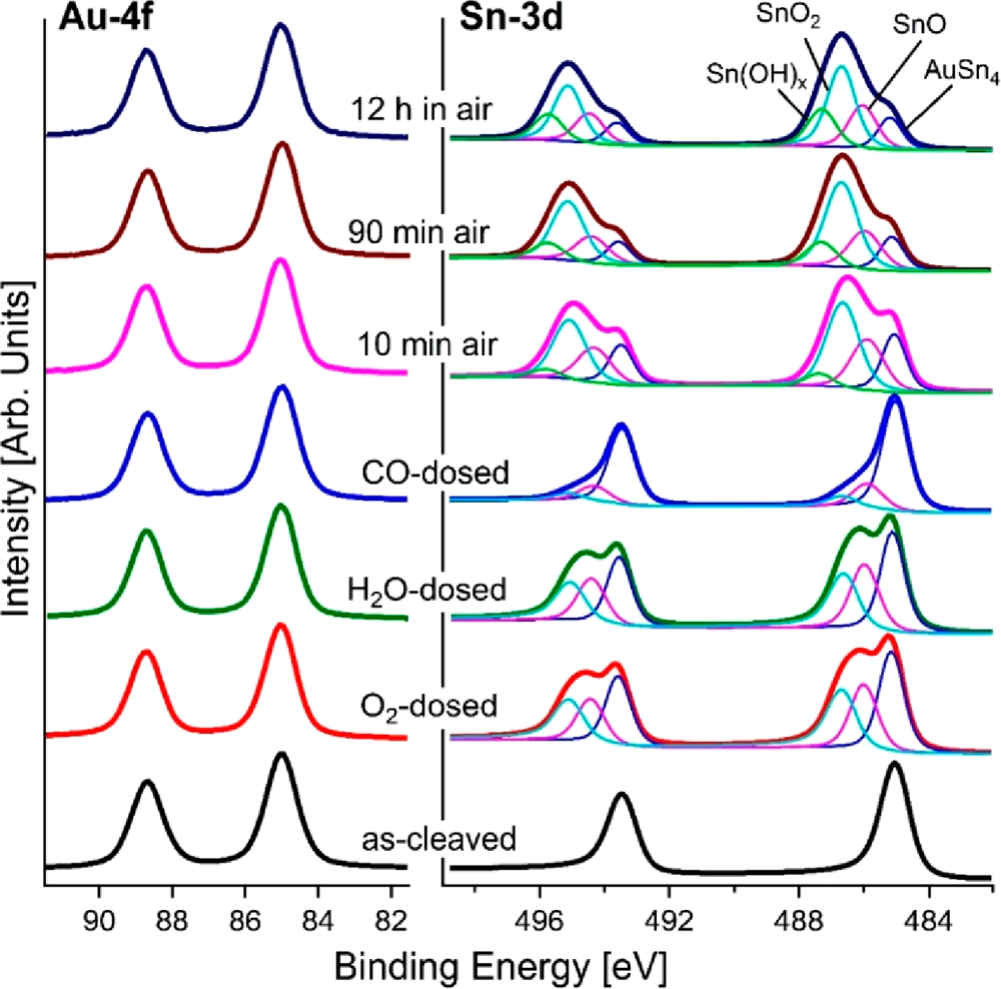
Core-level spectra of Au-4f and Sn-3d collected from as-cleaved AuSn_4_ (black curve) and from the same surface exposed to O_2_ (red curve), H_2_O (green curve), and CO (blue curve). The AuSn_4_ surface was also kept in air for different times: 10 min (pink curve), 90 min (brown curve), and 12 h (dark blue curve). The photon energy is 900 eV, and the spectra are normalized to the maximum.

On the other hand, with CO exposure a slight change in the spectra occurs with the formation of SnO (15% of the total spectral area) and SnO_2_ (6.5% of the total spectral area). The analysis of O-1s spectra (Figure S1 in Supporting Information) confirms the formation of oxide species for the O_2_- and H_2_O-dosed samples.

The as-cleaved AuSn_4_ was also directly exposed to air with the aim to assess its ambient stability. Au-4f and Sn-3d core-level spectra were measured as a function of exposure time to air to study the aging of AuSn_4_, as reported in Figure 5 (see also Supporting Information, Figure S1 for O-1s). The intensity of the oxide-derived components in the Sn-3d core levels gradually increased from ∼64% (after 10 min in air) to ∼76% (after 12 h in air) of the total spectral area. Specifically, after 10 min in air the formation of SnO, SnO_2_, and Sn(OH)_*x*_^29−33^ was evident in Sn-3d core levels, with a corresponding intensity of 23, 38, and 4% of the total spectral area, respectively. From 10 to 90 min in air, an enhancement of SnO_2_ (43% of the total spectral area) and Sn(OH)_*x*_ (11% of the total spectral area) components at the expense of SnO (20% of the total spectral area) was observed. After 12 h in air, hydroxylation of tin-oxide phases occurred, as indicated by the emergence of a Sn(OH)_*x*_ component (18% of the total spectral area).

By means of quantitative XPS analysis,^34^ we estimated the thickness of the oxide skin formed upon air exposure to be ∼2 nm, with passivation occurring in less than 15 min. The strong reactivity toward oxidation is also supported by considering that 10^4^ L O_2_ exposure in vacuum conditions already provided a oxide skin ∼1 nm thick.

To evaluate the effectiveness of AuSn_4_ for catalysis, we assessed its effectiveness for the hydrogen evolution reaction (HER), oxygen evolution reaction (OER), and CO_2_ reduction reaction (CO_2_RR).

For HER in acid media (Figure 6a), the first step of the model is the Volmer reaction:
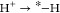
1i.e., the adsorption of hydrogen on the pristine and the oxidized (Figure 3d) surface. In the case of the pristine AuSn_4_ surface, hydrogen adsorption should overcome an energy barrier of +0.46 eV. The presence of Sn vacancies or of a tin-oxide skin turn the Volmer step to an exothermic process with an energy of −0.62 and −1.66 eV, respectively (Figure 6a). The formation of the oxide layer corresponds to visible changes in the subsurface Au layer. The adsorption of hydrogen on the tin-oxide surface corresponds to the partial reduction of the surface layer, resulting in a visibly affected electronic structure of the subsurface Au layer (see Figure 4e). In contrast, the formation of a Sn vacancy does not have a visible effect on the electronic structure of the nearest Au atom (Figure 4d), and adsorption of hydrogen does not correspond to a reduction of the surface layer since the energy of the Heyrovsky step of the HER in acidic media (2) has the same magnitude of energy of the Volmer step, but with an opposite sign.
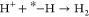
2Thus, for both AuSn_3.88_ and the oxidized AuSn_4_, the Heyrovsky step requires overcoming a significant energy barrier. Thus, the Tafel step is the only viable alternative.

**Figure 6:**
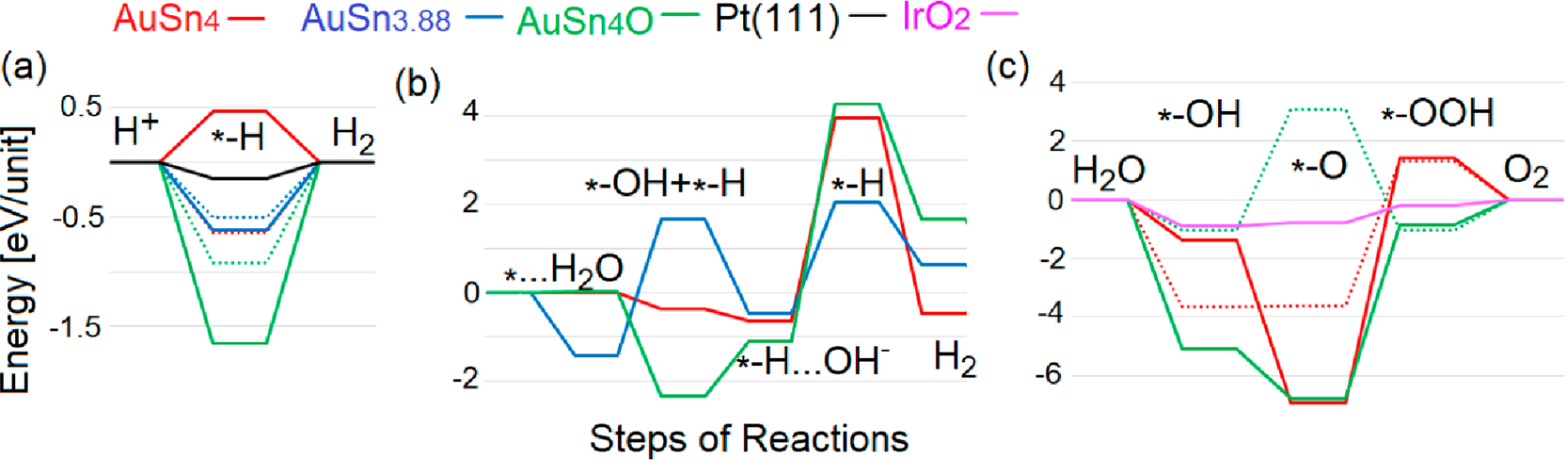
Free energy diagram for HER in (a) acidic and (b) alkali media and (c) OER in acidic media over AuSn_4_-based substrates and reference compounds. The oxidized surface of AuSn_4_ is denoted as AuSn_4_O. Dotted lines correspond to the energies of the same processes over similar PdSn_4_ substrates. The asterisk denotes the substrate, and “···” corresponds to physisorption.^35,36^

The Volmer step corresponds to the recombination of two hydrogen atoms adsorbed on the substrate:
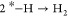
3

The rate of this reaction is typically limited by the energy barrier for the migration of hydrogen atoms across the substrate. The Volmer step is often overlooked due to its high energy barrier. However, the unique atomic structure of oxidized AuSn_4_ enables the migration of hydrogen atoms through the Sn–O layer with an energy cost of 0.19 eV. The breaking of the Sn–H bond (Figure 3d) is compensated by the interaction of hydrogen atoms with the electron cloud, which is distributed over the entire oxidized surface of AuSn_4_. As a result, the oxidized surface of AuSn_4_ is a favorable platform for HER in acidic media, due to the combination of the exothermic Volmer step and the low energy barrier for hydrogen atom migration over the surface.

**Table 2:** Calculated Gibbs Free Energies (in eV/mol) at Room Temperature of the Steps of CO_2_ Reduction over Pristine and Oxidized AuSn_4_ (Denoted as “ox-AuSn_4_”) Surface

step	reaction/substrate (*)	AuSn_4_	ox-AuSn_4_
1	H_2_ + * → 2(*-H)	0.98	–3.30
2	CO_2_ physisorption	0.83	0.35
3	CO_2_ + 2(*-H) → *-H + *-COOH	–1.20	–1.77
4	*-H + *-COOH → *-H + *–OH + *-CO	0.38	1.26
5	*-H + *-OH + *-CO → *-CO + *-H_2_O	-0.77	1.25
6	desorption of CO and H_2_O	-0.24	–0.93

The first step of the CO_2_RR involves the decomposition of molecular hydrogen on the surface of the catalyst. In the case of pristine AuSn_4_, an energy barrier of 0.98 eV/H_2_ exists, while for the oxidized AuSn_4_ this step is exothermic (−3.30 eV/H_2_), and, accordingly, molecular hydrogen will decompose. The next step of the reaction is the physisorption of carbon dioxide on the surface of the catalyst. For both considered surfaces, this step is endothermic, but the magnitude of the free energy required for adsorption on AuSn_4_ is more than twice as large than over the oxidized surface (0.83 vs 0.35 eV/CO_2_). Note that the energies required for physisorption of CO_2_ on the oxidized surface are just moderate. The third step of the CO_2_RR implicates the migration of the hydrogen atom adsorbed on the catalyst to carbon dioxide, with the subsequent transformation of the noncovalently adsorbed CO_2_ to −COOH or carboxyl groups, covalently adsorbed on the substrate. Contrary to ceria, where this step is endothermic,^35,36^ on AuSn_4_ it is exothermic for both pristine and oxidized surfaces. Such a difference is connected to the presence of electronic states around the Fermi level (Figure 1e–f). These topological features in the electronic structure correspond to outstanding electrical transport properties and also to chemical activity of the surface. In the case of an oxidized surface, topological features in the electronic structure of subsurface layers also affect the chemical activity of the oxidized surface layer via doping. The fourth step of the reaction corresponds to the migration of the −OH group from the carboxyl group to the surface of the catalyst. This step is endothermic for both substrates, but, in the case of an oxidized surface, the energy cost of this step is larger, because all suitable sites for oxidation spots on the surface would be already occupied. The fifth, penultimate, step of the process corresponds to the diffusion of the second hydrogen atom adsorbed at the surface of the catalyst toward the hydroxyl (−OH) group with the formation of physisorbed water molecules at the surface. In the case of the nonoxidized AuSn_4_ surface, the adsorption of hydrogen (first step of the reaction) and the hydroxyl group (fourth step of the reaction) is energetically unfavorable, resulting in the desorption of hydrogen from the surface and the favorable transformation of the hydroxyl group to water. In contrast, due to the strong adsorption of hydrogen atoms on the oxidized AuSn_4_, this step of the reaction is energetically unfavorable. The final step of the reaction involves the desorption of the products (carbon monoxide and water molecules) from the catalyst surfaces, which is energetically favorable for both substrates. Thus, the results of our calculations indicate that carbon dioxide reduction over AuSn_4_-based substrates involves alternating steps that are energetically favorable and unfavorable. For both substrates, the magnitudes of the values of free energies of exothermic steps overcome the energy costs of the endothermic steps. Note that the energy costs of the largest endothermic steps on both substrates (0.98 and 1.28 eV/mol) are of the same order (0.0–1.4 eV/mol) as those calculated for other prospective catalysts,^35,36^ including platinum.^37^ Thanks to the presence of topological features in the band structure that turn the rate-determining step of reaction to exothermic, both pure and oxidized AuSn_4_ can be considered as promising catalysts for this reaction. Moreover, it is worth noting that the already-oxidized surface of AuSn_4_ is expected to remain stable over time, unlike the nonoxidized surface, which is inherently unstable in air.

In conclusion, we have established a correlation between the exceptional catalytic activity of AuSn_4_ in the HER and CO_2_RR and the presence of topological electronic states. Contrary to previous reports, our model also considers the natural evolution of the catalyst’s surface upon interaction with air and finds that surface oxidation is even beneficial for the HER. We demonstrate that the combination of the exothermic Volmer step and the low energy barrier for the migration of hydrogen atoms over the surface makes the oxidized surface of AuSn_4_, a topological nodal-line semimetal, a suitable platform for HER in acidic media. Specifically, the theoretical model points out that the desorption of hydroxyl groups is the rate-determining step of HER in alkali conditions. For the oxidized AuSn_4_ surface, the first steps of the HER in alkali media are exothermic with rather large magnitudes of the energies, which could partially compensate the energy cost of the desorption of the hydroxyl groups. Hence, oxidized AuSn_4_ could be also considered as a catalyst of the HER in alkali media. Regarding CO_2_RR, we find that AuSn_4_ is a promising catalyst, due to the presence of topological electronic states. Specifically, the migration of the hydrogen atom adsorbed on rgw catalyst to carbon dioxide, with the subsequent conversion of the noncovalently adsorbed CO_2_ to −COOH or carboxyl groups, is exothermic for both pristine and oxidized surfaces of AuSn_4_. This is in contrast to other catalysts, including ceria, and is due to the presence of topological electronic states near the Fermi level, providing efficient electrical transport and subsequent superior chemical activity of the surface. In the case of the oxidized surface, the topological features in the electronic structure of subsurface layers also affect the chemical activity of the oxidized surface layer via doping.

Our results are significant in bringing quantum materials to fruition in catalysis, including the unforeseen role played by surface oxidation. Moreover, our findings can be applied to other materials with analogous physicochemical and structural properties.

Overall, our results shed light on the physicochemical mechanisms underlying catalytic reactions over the surfaces of quantum materials in the presence of an oxidizing ambient atmosphere. These findings also open new avenues for the design of high-performance catalysts based on topological nodal-line semimetals.

## Methods

*Single-Crystal Growth and Cleavage*. Single crystals of AuSn_4_ were synthesized by the self-flux method. High-purity Au (99.99%) and Sn ingots (99.999%) were sealed in an evacuated quartz tube with a flat bottom. The mixed elements were heated for 6 h, dwelled for 10 h, quickly cooled to 350 °C in 5 h, and then slowly cooled at a rate of 1 °C/h to 250 °C. Subsequently, the excess Sn flux was removed by centrifugation and then etched in concentrated hydrochloric acid. The AuSn_4_ crystals were cleaved in a ultrahigh vacuum by a postmethod with a natural cleavage plane coinciding with the (010) orientation.

*ARPES Measurements*. ARPES measurements on the AuSn_4_ single crystals were performed at the APE-LE beamline of ELETTRA Synchrotron in Trieste, Italy, using a DA30 electron energy analyzer. The spectra presented here were acquired with 48 eV photon energy.

*XPS Measurements*. XPS experiments were performed at the High-Energy branch of the Advanced Photoelectric Experiments beamline (APE-HE) of the Elettra Synchrotron, Trieste, Italy. XPS spectra were acquired with an Omicron EA125 hemispherical electron energy analyzer, with the sample at room temperature and in normal emission conditions. The linearly polarized light was impinging on the sample forming an angle of 45 deg with respect to the normal to the surface. Under our experimental conditions, there is no beam-induced damage.

*DFT Calculations*. The atomic structure and energetics of various configurations was studied by DFT using the QUANTUM-ESPRESSO code^38^ and the GGA–PBE,^39^ taking into account van der Waals forces correction.^40^ For all calculations, we used ultrasoft pseudopotentials.^41^ The values of energy cutoffs were 25 and 400 Ry for the plane-wave expansion of the wave functions and the charge density, respectively. The possible influence of correlation effects on the electronic structure of AuSn_4_ was checked by DFT+U calculations, which demonstrated their negligible influence on the band structure in the vicinity of the Fermi energy (see Figure S2).

The enthalpy of reaction is defined as difference in calculated total energies of products and reactant. Thus, negative enthalpy corresponds to exothermic reactions.

Physisorption enthalpies were calculated by the standard formula:
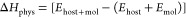
where *E*_host_ is the total energy of pristine surface, and *E*_mol_ is the energy of the single molecules of selected species in empty box. In the case of water adsorption, we only considered the gaseous phase. Decomposition energy is defined as difference between the total energy of the system with the adsorbed molecule and the total energy of same system after decomposition of the same molecule on the surface. For the case of physisorption, we also evaluated differential Gibbs free energy by the formula:
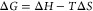
where *T* is the temperature and Δ*S* is the change of entropy of the adsorbed molecule, which was estimated considering the gas → liquid transition by the standard formula:
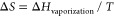
where Δ*H*_vaporization_ is the measured enthalpy of vaporization.

## Supporting Information Available

O-1s core-level spectra; band structure calculated with DFT+U (PDF)
