# Supplementary material for: Unveiling the Catalytic Potential of Topological Nodal-Line Semimetal AuSn4 for Hydrogen Evolution and CO2 Reduction
Source: J Phys Chem Lett. 2023 Mar 22;14(12):3069–76. doi: 10.1021/acs.jpclett.3c00113 (PMC10068825; doi:10.1021/acs.jpclett.3c00113)
Supplement: Supplementary file 1 — jz3c00113_si_001.pdf [file jz3c00113_si_001.pdf]

SUPPORTING INFORMATION

Unveiling the Catalytic Potential of Topological Nodal-Line Semimetal $AuSn_4$ for Hydrogen Evolution and CO_2 Reduction

Danil W. Boukhvalov[1,2], Gianluca D'Olimpio[3], Federico Mazzola[4], Chia-Nung Kuo[5], Sougata Mardanya[5], Jun Fujii[4], Grazia Giuseppina Politano[6], Chin Shan Lue[5], Amit Agarwal[7], Ivana Vobornik[4], Piero Torelli[4], Antonio Politano[3]

[1] College of Science, Institute of Materials Physics and Chemistry, Nanjing Forestry University, Nanjing 210037, P. R. China
[2] Institute of Physics and Technology, Ural Federal University, Mira Str. 19, 620002 Yekaterinburg, Russia
[3] Department of Physical and Chemical Sciences, University of L'Aquila, via Vetoio, 67100 L'Aquila (AQ), Italy
[4] Consiglio Nazionale delle Ricerche (CNR)- Istituto Officina dei Materiali (IOM), Laboratorio TASC in Area Science Park S.S. 14 km 163.5 34149 Trieste, Italy
[5] Department of Physics, National Cheng Kung University, 1 Ta-Hsueh Road, 70101 Tainan, Taiwan
[6] Department of Information Engineering, Infrastructures and Sustainable Energy (DIIES), University "Mediterranea" of Reggio Calabria, Loc. Feo di Vito, 89122 Reggio Calabria, Italy
[7] Department of Physics, Indian Institute of Technology Kanpur, Kanpur 208016, India.

S1. O-1s core level of $AuSn_4$ before and after exposure to O_2, CO, H_2O and air

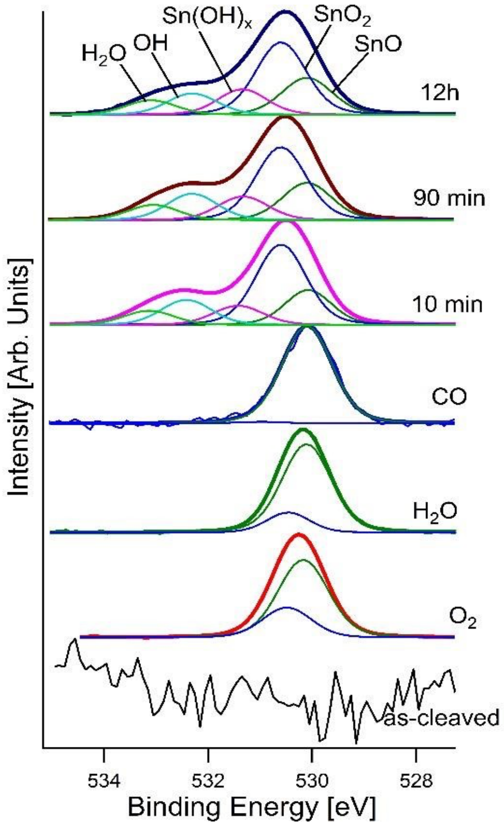

Figure S1: *O-1s Core-level spectra collected from as-cleaved $AuSn_4$ (black curve) and from the same surface exposed to O_2 (red curve), H_2O (green curve) and CO (blue curve). The $AuSn_4$ surface was also exposed to air for different times, 10 minutes (pink curve), 90 minutes (brown curve) and 12 hours (dark blue curve). The photon energy is 900 eV and the spectra are normalized to the maximum.*

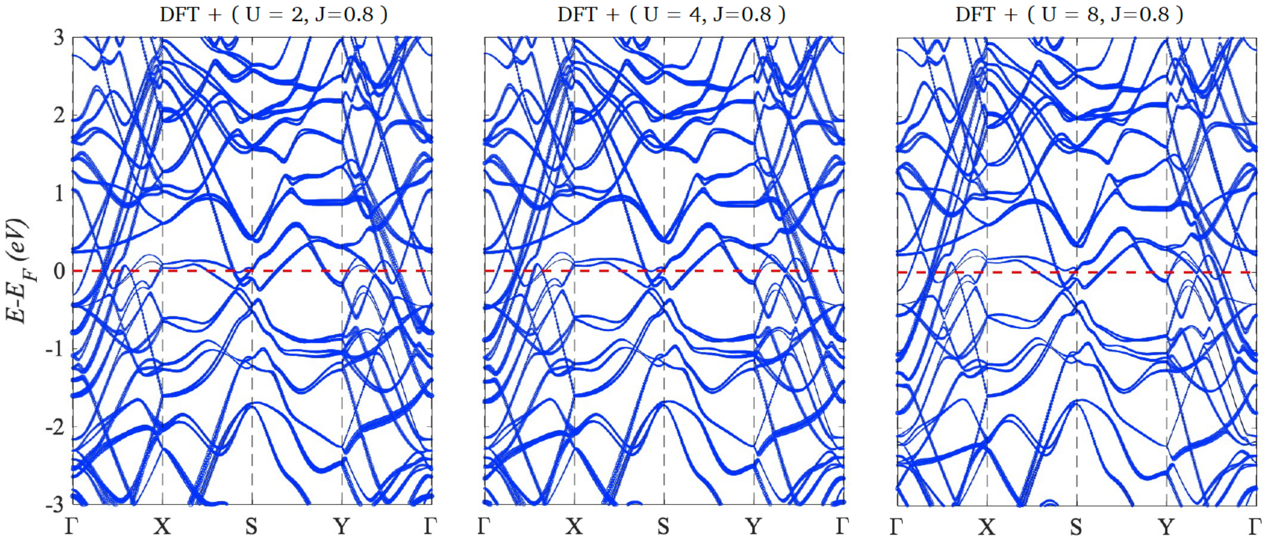

Figure S2: *Band structure calculated with DFT+U, with U=2, 4, 8 eV*
